# Supplementary material for: A procedure for risk assessment of check dam systems: A case study of Wangmaogou watershed
Source: PLoS One. 2023 Jun 27;18(6):e0287750. doi: 10.1371/journal.pone.0287750 (PMC10298768; doi:10.1371/journal.pone.0287750)
Supplement: Attachment — Submitted filename: comment 9.2.docx [file pone.0287750.s004.docx]

Check dams are applied worldwide as an effective approach for soil conservation, However, failure risk of check dams restricts its development. The paper proposes a weighting method that combines the analytic hierarchy process and entropy method with TOPSIS to assess the risk of check dam systems. Although the research method of the paper is not innovative enough, the process and application of the method are introduced clearly, the content is rich, and it is valuable. Therefore, I recommend accepting this manuscript for publication, after some concerns are clarified with major revisions. My specific comments and suggestions are followed:

1. Manuscripts should be labeled with line numbers to help reviewers point out specific problems.
2. The research purpose of the paper should be introduced more clearly, and the conclusion should be more concise.
3. Why choose check dam system unit for evaluation instead of check dam system or single check dam?
4. Supplement the dividing standard of check dam system unit.
5. The evaluation results of check dam system units should be verified by actual investigation.
