# Supplementary material for: A procedure for risk assessment of check dam systems: A case study of Wangmaogou watershed
Source: PLoS One. 2023 Jun 27;18(6):e0287750. doi: 10.1371/journal.pone.0287750 (PMC10298768; doi:10.1371/journal.pone.0287750)
Supplement: Attachment — Submitted filename: Response to Reviewers.docx [file pone.0287750.s006.docx]

**Ref.: Manuscript Number: PONE-D-22-15836R2**

**Title: A Procedure for Risk Assessment of Check Dam Systems: A Case Study of Wangmaogou Watershed**

**Thanks for your positive comments. We have responded to Editor ’s question.**

Editor ’s Questions:

1. Thank you for your response. Please update the Figure captions of Figure 5 to include attribution to ESRI. Once this is done, we should be able to proceed.

Reply:

Thank you for your comment. The Figure caption of Figure 5 in Line 246, Page16 now has been modified to“Fig 5. Location of the area (a, b); the Wangmaogou watershed (c, Drawn by ESRI's ArcGIS); the layout of check dam types (d).”
