# Supplementary material for: A procedure for risk assessment of check dam systems: A case study of Wangmaogou watershed
Source: PLoS One. 2023 Jun 27;18(6):e0287750. doi: 10.1371/journal.pone.0287750 (PMC10298768; doi:10.1371/journal.pone.0287750)
Supplement: Attachment — Submitted filename: Response to Reviewers.docx [file pone.0287750.s007.docx]

**Ref.: Manuscript Number: PONE-D-22-15836R2**

**Title: A Procedure for Risk Assessment of Check Dam Systems: A Case Study of Wangmaogou Watershed**

**Comments to the Author**

**4. Have the authors made all data underlying the findings in their manuscript fully available?**

The [PLOS Data policy](http://www.plosone.org/static/policies.action#sharing) requires authors to make all data underlying the findings described in their manuscript fully available without restriction, with rare exception (please refer to the Data Availability Statement in the manuscript PDF file). The data should be provided as part of the manuscript or its supporting information, or deposited to a public repository. For example, in addition to summary statistics, the data points behind means, medians and variance measures should be available. If there are restrictions on publicly sharing data—e.g., participant privacy or use of data from a third party—those must be specified.

Reviewer #2: No

Reviewer #3: Yes

Reply:

Thank you for your comment.

All the data has been uploaded to <https://github.com/XAUT-WangLin/Summary-table-of-evaluation-index>. It includes 1 copy of the Wangmaogou DEM file, 1 copy of Summary table of evaluation index of Wangmaogou watershed, and 1 copy of calculating table. These data completely include the topographic data used in the paper, the raw data used in the calculation of the paper, and the data of the whole process of the thesis operation. The data is open source and can be downloaded and used via the connection above.

**6. Review Comments to the Author**

Please use the space provided to explain your answers to the questions above. You may also include additional comments for the author, including concerns about dual publication, research ethics, or publication ethics. (Please upload your review as an attachment if it exceeds 20,000 characters)

Reviewer #2: (No Response)

Reviewer #3: Author should correct a few identified typographical and grammatical errors noticed in the manuscript. For instance, on line 38, a word lagre is supposed to be large.

Reply:

Thanks for your comments. We have revised manuscript carefully and the details are as follws:

In Line 38, “lagre” has been corrected to “large”.

In Line 320, “The” has been corrected to “the”.

In Line 390, “Huangbogou” has been corrected to “Huangbaigou”.

All the “Kang Hegou” has been corrected to “Kanghegou”.
