# Supplementary material for: A procedure for risk assessment of check dam systems: A case study of Wangmaogou watershed
Source: PLoS One. 2023 Jun 27;18(6):e0287750. doi: 10.1371/journal.pone.0287750 (PMC10298768; doi:10.1371/journal.pone.0287750)
Supplement: Attachment — Submitted filename: Response to Reviewers.docx [file pone.0287750.s008.docx]

**Ref.: Manuscript Number: PONE-D-22-15836R3**

**Title: A Procedure for Risk Assessment of Check Dam Systems: A Case Study of Wangmaogou Watershed**

Additional Editor Comments:
This manuscript is acceptable, however, substantial editing in terms of formatting, and improvement in sentence structuring need to be improved. Also make sure that paragraphs are well aligned. Further, please remove the figure caption's writing that author owns the copy right. If author burrowed then they should write the source otherwise no need to write copy right. Also, most figure caption requires little bit more information to elaborate figure caption. Thank you for revision.

reply:

Thanks for your positive advice and we are so glad to receive the message that you would like to accept this manuscript. We also revise our manuscript according to your letter, and the details are as follow:

1. The figure caption's writing that author owns the copy right now has been removed from Fig.1, Fig.2 and Fig.8.
2. The Fig.5 caption now has been revised as: “Fig 5. Location of the area (a, b); tthe altitude of the Wangmaogou watershed (c, Drawn by ESRI's ArcGIS); the layout of check dam types (d).”
3. Some words and the figure size has been adjusted to make Table.1 and Table.3 more suitable for pages.
